# Sero-prevalence of syphilis and associated factors among pregnant women in Ethiopia: a systematic review and meta-analysis

**DOI:** 10.1186/s13643-021-01786-3

**Published:** 2021-08-12

**Authors:** Habtamu Geremew, Demeke Geremew

**Affiliations:** 1 Department of Public Health, College of Health Sciences, Debre Markos University, Debre Markos, Ethiopia; 2 Department of Immunology and Molecular Biology, School of Biomedical and Laboratory Sciences, University of Gondar, P.o.Box: 196, Gondar, Ethiopia

**Keywords:** Syphilis, Aero-prevalence, Pregnant women, Meta-analysis, Ethiopia

## Abstract

**Background:**

Syphilis remained a major cause of reproductive morbidity and poor pregnancy outcomes in developing countries. Previously, studies showed inconsistent results and failed to show the actual picture of the diseases in Ethiopia. Thus, the aim of this meta-analysis was, first, to determine the updated pooled prevalence of syphilis among pregnant women in Ethiopia and, second, to assess its associated factors.

**Methods:**

A comprehensive search was made on PubMed, Google scholar, Science Direct, and African Journals Online databases to identify relevant articles. A random effects model was used to estimate pooled syphilis prevalence and odds ratio (OR) with the respective 95% confidence intervals (CIs) using STATA 14 statistical software. *I*^2^ statistics and Egger’s regression test in conjunction with funnel plot was used to determine heterogeneity and publication bias among included studies respectively.

**Result:**

We identified 13 suitable studies in this analysis. Accordingly, the pooled prevalence of syphilis among pregnant women in Ethiopia was 2.32% (95% CI, 1.68–2.97). Specifically, syphilis prevalence was 2.53% (95% CI, 1.92–3.14%) and 1.90% (95% CI, 0.40–3.40%) as per the treponemal and non-ytreponemal diagnostic test, respectively. On the other hand, regional analysis indicated that 4.06% (95% CI, 2.86–5.26) in Southern Nations Nationalities and Peoples (SNNP), 2.16% (95% CI, 1.57–2.75) in Amhara and 1.46% (95% CI, 0.69–2.23) in Oromia region. Being married (OR, 0.37 (95% CI, 0.12–0.91%)) was less likely to develop syphilis. On the other hand, women with history of multiple sexual partner (OR, 2.98 (95% CI, 1.15–7.70)) and women with history of previous sexually transmitted infection (STI) (OR, 4.88 (95% CI, 1.35–17.62)) have higher risk to develop syphilis. Besides, the pooled syphilis-HIV coinfection was 0.80% (95% CI, 0.60–1.01%).

**Conclusion:**

This study provides evidence of relatively high prevalence of syphilis among pregnant women in Ethiopia. Therefore, it is recommended to further ramping up of current intervention measures to prevent future generations.

**Systematic review registration:**

PROSPERO CRD42020211650

**Supplementary Information:**

The online version contains supplementary material available at 10.1186/s13643-021-01786-3.

## Background

Syphilis is a sexually transmitted infection (STI) caused by the spirochete *Treponema pallidum*, and it continues to be a main public health problem worldwide [1]. It spreads primarily through sexual contact and vertical transmission and can rarely be spread through blood transfusion. Syphilis can be successfully controlled by effective public health measures due to the availability of a sound diagnostic test and effective and economical treatment options [2]. However, if syphilis is left untreated, it can lead to devastating fetal outcomes [3].

Pregnant women are sexually active and are at risk of STI, including syphilis [4]. Globally, 36 million people are infected with syphilis, out of which 2 million are pregnant women. More than half of infected women transmit the infection to their babies resulting in adverse pregnancy outcomes including early fetal death, stillbirth, preterm birth, low birth weight, neonatal death, and congenital infection in infants [5].

Syphilis remained a major cause of reproductive morbidity and poor pregnancy outcomes in developing countries [4]. In sub-Saharan Africa, syphilis sero-prevalence ranges from 4 to 15%, and can cause adverse outcomes in 50–80% of pregnancies [6]. Ethiopia is among the top three sub-Saharan countries with the highest numbers of adverse pregnancy outcomes attributed to syphilis [7]. Furthermore, studies have demonstrated that 21% children born from seropositive mothers in Ethiopia developed signs of syphilis. Besides, stillbirth and abortion rates of syphilis diagnosed women were almost double relative to the general population [8].

Previously established systematic review and meta-analysis conducted on the prevalence of syphilis among pregnant women in Ethiopia includes only five studies with smaller sample size and did not report syphilis prevalence based on the diagnostic test modality. Besides, it failed to demonstrate syphilis-HIV co-infection and predictors of syphilis sero-positivity [9]. Thus, in the absence of concrete and inclusive evidence in STI endemic settings including HIV and syphilis, this systematic review and meta-analysis was conducted to determine the updated pooled prevalence of syphilis among pregnant women in Ethiopia. Moreover, syphilis sero-reactivity associated factors and syphilis-HIV co-infection was also determined in this study to guide public health intervention and control measures.

## Methods and materials

### Reporting and study protocol registration

This review was conducted according to the requirements of the Preferred Reporting Items for Systematic Reviews and Meta-Analyses (PRISMA) statement [10] (Additional file 1). This study was registered in International Prospective Register of Systematic Reviews (PROSPERO) database with protocol number, CRD42020211650.

### Search strategy and information sources

An inclusive literature search was made from September 1 to 30, 2020, on PubMed, Google scholar, Science Direct, and African Journals Online databases based on Preferred Reporting Items for Systematic Reviews and Meta-Analyses (PRISMA) statement [10]. The following key words were used for PubMed database searching: [“Syphilis” or “Treponema pallidum” AND (magnitude OR prevalence OR seroprevalance) AND “pregnant women” OR “pregnant” AND “Ethiopia”]. In addition, literatures and reference lists of relevant articles were also retrieved to find additional studies.

### Inclusion and exclusion criteria

All articles fulfilling the following conditions were screened and subsequently assessed for eligibility. Published studies conducted only in Ethiopia and reporting prevalence of syphilis among pregnant women, published in English language up to the end of September 2020. Studies with a clear description of participants’ involved and state the number of participants tested for syphilis, and articles with a clear number and/or prevalence of syphilis cases were also considered. Nevertheless, citations without abstracts and/or full-text, review articles, conference abstracts, editorials, duplicate studies, commentaries, trend analyses, studies which include non-pregnant women and those which do not report syphilis prevalence were excluded from the review. Similarly, given the known synergy between HIV and syphilis [11], studies conducted prior to HIV antiretroviral availability (before January 2005 in Ethiopia) [12] were also excluded as this would affect syphilis prevalence.

### Outcome of interest

The primary outcome of this study was the prevalence of syphilis among pregnant women. Moreover, the results have been stratified by diagnostic test (treponemal vs non-treponemal testing) and different geographical regions in Ethiopia. Secondly, we have also determined factors associated with syphilis sero-positivity: marital status (married vs not cohabiting; not cohabiting involves: single, widowed, and divorced), previous STI (present vs absent), and previous history of multiple sexual partner (present vs absent). In addition, the prevalence of syphilis-HIV co-infection was also determined.

### Study selection, quality assessment, and data extraction

The title and abstract of studies were screened after removing duplicates. Full-text review was conducted for articles found to be relevant by tittle and abstract to identify potential articles for inclusion in this meta-analysis.

The quality of included studies was evaluated by using Joanna Brigg’s Institute (JBI) quality assessment checklist for prevalence studies [13]. Based on the JBI checklist, studies with a quality score of 50% and above were considered high quality and involved in the analysis. Extracted data includes the following descriptive information: author and publication year, study area/region, study period, laboratory methods employed to diagnose syphilis, sample size, and prevalence of syphilis. In addition, prevalence of syphilis by marital status, presence/absence of previous history of multiple sexual partner, and history of previous STI were extracted whenever reported. Two independent reviewers (HG and DG) were involved in study selection, quality assessment, and data extraction. Disagreement between the reviewers was solved by discussion.

### Statistical methods and analysis

Data were entered into Microsoft Excel and then exported to STATA 14 statistical software for further analysis. The *I*^2^ values of 25, 50, and 75% was considered low, medium, and high heterogeneity, respectively [14]. In pooled prevalence analysis and 95% confidence intervals (CIs), the random effects model (DerSimonian-Laird method) [15] was used. The overall and subgroup analysis of random effects model with 95% CIs were calculated and demonstrated using a forest plot. Nevertheless, for studies with small (near 0) or large (near 1) prevalence, the inverse variance method was not stable and hence, we used Freeman Tukey arcsine methodology to address stabilizing variances as evidenced elsewhere [12, 16, 17]. Syphilis-HIV co-infection and the effect of selected predictor variables including marital status, previous history of multiple sexual partner, and previous history of STI on syphilis prevalence was analyzed using separate categories of meta-analysis.

The existence of publication bias was determined using funnel plot and Egger’s regression test. In Egger’s test, *p* < 0.05 was considered statistically significant [18]. The effect of each study on the overall pooled prevalence was determined by using sensitivity analysis. Sensitivity test eliminates each study step by step in the analysis to indicate the pooled effect sizes and related heterogeneity attributed by each individual study.

## Result

### Study selection

**Fig. 1:**
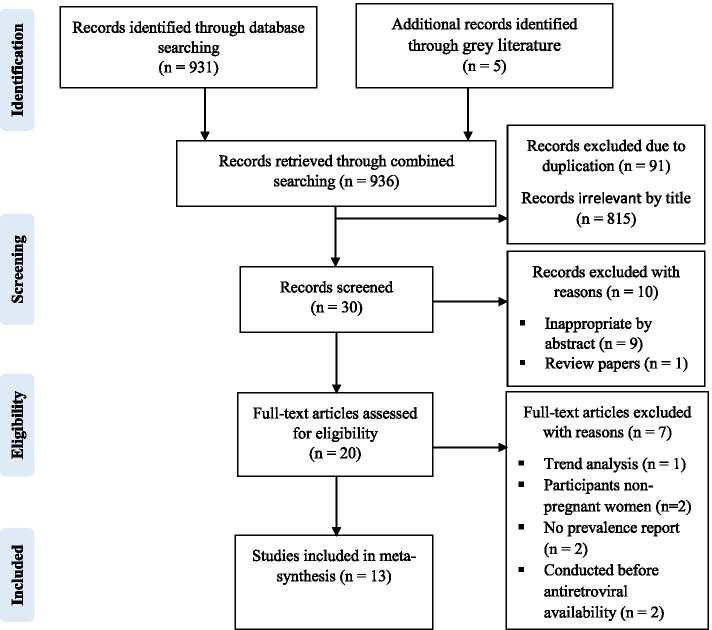
PRISMA flow chart for the studies screened, reviewed and included

A comprehensive combined literature search generated a total of 936 possible articles, of which 20 were chosen sensibly for detailed full-text assessment and 13 studies were found to be appropriate for consideration in the meta-synthesis (Fig. 1).

### Characteristics of included studies

Among the 13 studies [19–31] included in the meta-analysis, a total of 17,656 pregnant women were screened for syphilis in three different regions of Ethiopia. Regional distribution of studies revealed that eight [19–24, 26, 29] from Amhara, two [25, 28] from Southern Nations Nationalities and Peoples (SNNP) and three [27, 30, 31] from Oromia region. In spite of that, there was no any study from other regions of Ethiopia fulfilling the inclusion criteria. Except a single prospective cohort study [27] which was conducted in Assela, Oromia region; all included records were cross-sectional studies with study participants ranging from 181 [31] to 4346 [30] and conducted from 2005 to 2019. Different diagnostic tests were employed for screening syphilis; six studies [19–22, 25, 31] used treponema pallidum hemagglutination assay (TPHA), three studies [24, 28, 29] used venereal diseases research laboratory (VDRL), two studies [26, 30] employed rapid plasma reagin (RPR) test, and one study [27] used syphilis rapid immunoassay, while the other one [23] used Immuno-chromatography test strips (ICS test). As per the established literature, the TPHA, syphilis rapid immunoassay and ICS test constitute the treponemal test while the VDRL and RPR are non-treponemal diagnostic tests [32].

Out of 17,656 pregnant women screened for syphilis, 416 were found positive. Out of 416 seropositive women, 172 were from SNNP, 183 were from Amhara, and 61 were from Oromia (Table 1).

**Table 1:** Summary characteristics of studies included in the meta-analysis

Author and year	Study area/region	Study period	Sample size	Test used	Prevalence of syphilis	Quality assessment
Mulu et al., 2007 [26]	Gondar/Amhara	March to June, 2005	480	RPR	1%	High quality
Abate Assefa, 2014 [19]	Gondar/Amhara	January 2009 to December 2011	2385	TPHA	2.9%	High quality
Endris et al., 2015 [21]	Gondar/Amhara	February to June 2011	385	TPHA	2.9%	High quality
Melku et al., 2015 [22]	Gondar/Amhara	March to May 2012	300	TPHA	3.7%	High quality
Schonfeld et al., 2017 [27]	Assela/Oromia	May 2014 to September 2015	580	SRIA*	2.2%	High quality
Ageru et al., 2018 [28]	Wolaita Sodo/SNNP	30 June 2012 to 30, June 2016	4022	VDRL	3.7%	High quality
Amsalu et al., 2018 [25]	Yirgalem/SNNP	October 2015 to August 2016	494	TPHA	5.1%	High quality
Zinabie et al., 2018 [29]	Debre Berhan/Amhara	September 2015 to August 2017	385	VDRL	1.8%	High quality
Asfaw et al., 2019 [31]	Jimma/Oromia	January to June, 2016	181	TPHA	1.1%	High quality
Biadgo et al., 2019 [20]	Gondar/Amhara	January 2011 to April 2015	3504	TPHA	1.9%	High quality
Mekonnen et al., 2019 [30]	Shashemene/Oromia	January 2014 to December 2016	4346	RPR	1.1%	High quality
Tareke et al., 2019 [23]	Bahir Dar/Amhara	November 2013 to June 2014	384	ICS test	2.6%	High quality
Yitbarek et al., 2019 [24]	Sede Muja District/Amhara	November 2018 to January 2019	210	VDRL	1.9%	High quality

*ICS* immuno-chromatography strips, *RPR* rapid plasma regain, *SNNP* Southern Nations Nationalities and Peoples of Ethiopia, *TPHA* treponema pallidum hemagglutination assay, *VDRL* venereal diseases research laboratory, SRIA* syphilis rapid immunoassay test

### Prevalence of syphilis among pregnant women

**Fig. 2:**
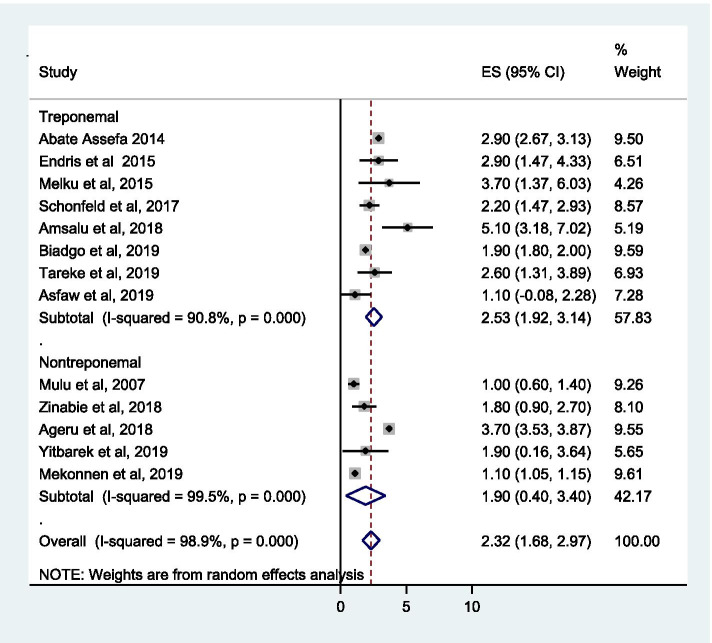
Pooled prevalence estimate (ES) of syphilis among pregnant women in Ethiopia

**Fig. 3:**
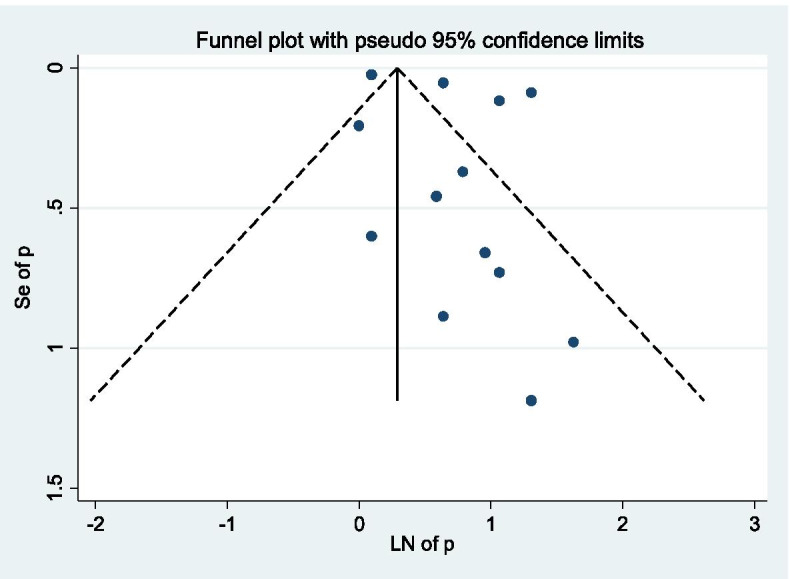
Funnel plot, evaluating the existence of publication bias for pooled syphilis prevalence

The pooled prevalence of syphilis among pregnant women in Ethiopia from the random effects model was 2.32% (95% CI, 1.68–2.97; *I*^2^ = 98.9%; Eggers test, *p* = 0.14). Subgroup analysis based on the diagnostic test employed indicated that 2.53% (95% CI, 1.92–3.14%) and 1.90% (95% CI, 0.40–3.40%) syphilis prevalence using treponemal and non-treponemal test, respectively (Fig. 2). As graphically demonstrated in the symmetrical funnel plot, there was no evidence of publication bias within included studies (Fig. 3). Moreover, this was also assured by Egger’s test (*p* = 0.14). In addition, the sensitivity analysis clearly indicated that the influence of individual studies on the summary effect estimate was not significant. Consequently, the pooled effect size estimate of syphilis among pregnant women in Ethiopia was steady and reliable when analyzed by omitting one study at a time (Table 2).

**Table 2:** Sensitivity analysis

Excluded studies	Pooled prevalence (95% CI)
Mulu et al., 2007 [26]	2.46 (1.77, 3.14)
Abate Assefa., 2014 [19]	2.26 (1.59, 2.93)
Endris et al., 2015 [21]	2.28 (1.62, 2.95)
Melku et al., 2015 [22]	2.26 (1.60, 2.92)
Schonfeld et al., 2017 [27]	2.33 (1.66, 3.01)
Amsalu et al., 2018 [25]	2.17 (1.51, 2.83)
Zinabie et al., 2018 [29]	2.37 (1.69, 3.04)
Ageru et al., 2018 [28]	2.09 (1.59, 2.59)
Biadgo et al., 2019 [20]	2.41 (1.50, 3.33)
Tareke et al., 2019 [23]	2.30 (1.63, 2.97)
Yitbarek et al., 2019 [24]	2.35 (1.68, 3.01)
Mekonnen et al., 2019 [30]	2.45 (1.79, 3.11)
Asfaw et al., 2019 [31]	2.42 (1.75, 3.09)
Combined	2.32 (1.68, 2.97)

### Syphilis prevalence in different regions of Ethiopia

The prevalence of syphilis based on the geographical regions of Ethiopia was 4.06% (95% CI, 2.86–5.26) in SNNP, 2.16% (95% CI, 1.57–2.75) in Amhara, and 1.46% (95% CI, 0.69–2.23) in Oromia region (Table 3).

**Table 3:** Summary estimate for syphilis prevalence in different regions of Ethiopia

Geographical regions	Included studies	Prevalence (95% CI)	*I*^2^
Amhara	8	2.16% (95% CI, 1.57–2.75)	92.2%
Oromia	3	1.46% (95% CI, 0.69–2.23)	77.2%
SNNP	2	4.06% (95% CI, 2.86–5.26)	50.6%

*SNNP* Southern Nations Nationalities and Peoples of Ethiopia

### Pooled prevalence of syphilis-HIV co-infection among pregnant women

**Fig. 4:**
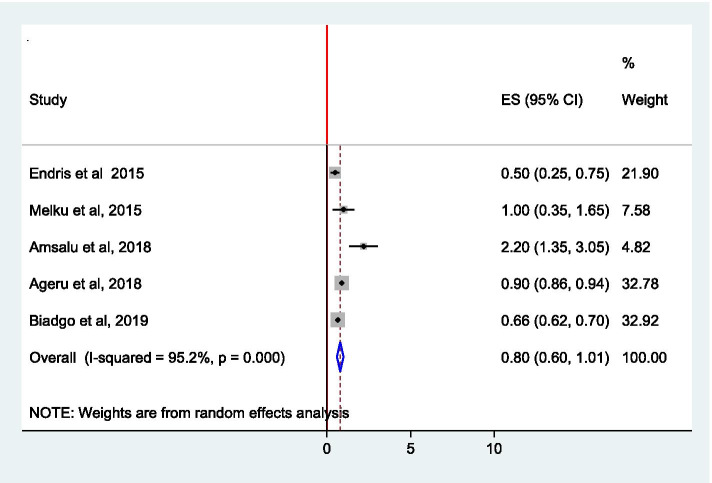
Pooled prevalence estimate (ES) of syphilis-HIV co-infection among pregnant women in Ethiopia

The overall pooled prevalence of syphilis-HIV co-infection among pregnant women in this meta-analysis was 0.80% (95% CI, 0.60–1.01; *I*^2^ = 95.2%) (Fig. 4).

### Factors associated with syphilis sero-positivity

The association of syphilis sero-positivity with marital status, previous history of multiple sexual partner and previous history of STI was quantified using OR. The odds of having syphilis are almost three times higher in pregnant women who had history of multiple sexual partners than those who had no such partner, OR 2.98 (95% CI, 1.15–7.70). Pregnant women who had previous history of STI were more than four times more likely to develop syphilis than women who had no such history, OR 4.88 (95% CI, 1.35–17.62), whereas married women had 63% lower risk of having syphilis compared to those who are not cohabiting (single, widowed, divorced), OR 0.37 (95% CI, 0.12–0.91) (Table 4).

**Table 4:** Summary estimate of OR for syphilis sero-positivity associated factors

Variables	Intervention	Comparator	Included studies	OR (95% CI)	*I*^2^
Multiple sexual partner history	Present	Absent	2	2.98 (1.15–7.70%)	15.8%
Marital status	Married	Not cohabiting	5	0.37 (0.12–0.91%)	60.2%
Previous history of STI	Present	Absent	2	4.88 (1.35–17.62%)	17.1%

*STI* sexually transmitted infection

## Discussion

Syphilis is one of the easily preventable and treatable sexually transmitted infections but continued to exert a high burden worldwide especially in sub-Saharan Africa where resources are scarce. Therefore, prevention and control of syphilis among pregnant women using appropriate intervention measures is crucial for the emergence of syphilis free generation. For that, determining the actual burden of syphilis infection and antenatal care (ANC) follow-up screening plays a great role in early diagnosing and treatment of syphilis, and prevention of its vertical transmission. This study aimed to determine the updated pooled prevalence and factors associated to syphilis positivity, and the pooled syphilis-HIV co-infection among pregnant women in Ethiopia.

This meta-analysis showed that the overall pooled prevalence of syphilis among pregnant women in Ethiopia was 2.32% (95% CI, 1.68–2.97). The pooled prevalence was two times higher than the recent nationwide HIV/syphilis sentinel reports among ANC attendees in Ethiopia (1.1%) [33]. The variation in syphilis prevalence between this meta-analysis and the sentinel survey might be due to the rough estimate nature of the sentinel report that might underrepresent the actual burden of the diseases. Contrastingly, previous meta-analysis reported higher prevalence of syphilis among pregnant women in Ethiopia, 3.67% [9] relative to this study. This might be because of the previous meta-analysis includes studies with higher syphilis prevalence conducted in the pre-antiretroviral therapy availability. However, given the known synergy between HIV and syphilis [11], we have excluded studies conducted prior to HIV antiretroviral availability in this meta-analysis. Besides, the diseases dynamics may change overtime and impact the observed prevalence [34]. Partly, it may be due to the difference in the number of included studies (5 studies in earlier meta-analysis and 13 articles in this study), indicating the sample size may impact the prevalence.

On the other hand, the pooled prevalence in this meta-analysis was comparable with the report from a countrywide surveillance of HIV/syphilis prevalence among pregnant women attending ANC in Tanzania (2.5%) [35]. The similarity in syphilis prevalence in Ethiopia and Tanzania might be partly due to the WHO’s increased focus and prioritization of antenatal syphilis (in conjunction with HIV and hepatitis B virus) screening for better intervention measures. Besides, combination rapid HIV/syphilis tests are now used to a greater degree in ANC which may also result in increased syphilis testing/diagnoses in different countries of the world to indicate the actual picture of the diseases in various settings.

As shown in subgroup analysis, this study indicated relatively higher prevalence of syphilis using treponemal diagnostic test modality 2.53% (95% CI, 1.92–3.14%) compared to the non-treponemal test 1.90% (95% CI, 0.40–3.40%). This could be due to reactivity to a treponemal test implies infection but it does not determine whether the infection is recent or remote or whether it has been treated or not [32]. Thus, it suggests that treponemal tests stay positive for decades after treatment and may not always indicate active infection. On the other hand, non-treponemal tests have a high false-positive rate and are difficult to interpret on their own [32]. Therefore, considering the difficulty of syphilis diagnosis, the results have to be interpreted with care.

Regional analysis showed a higher and lower prevalence of syphilis in SNNP (4.06%) and in Oromia (1.46%) respectively. Higher syphilis prevalence in SNNP might be attributed to the risky socio-cultural practices such as polygamy is more practiced in SNNP [36] relative to other regions of Ethiopia. Partly, it might be due to the difference in the number of studies included in each category.

In addition, pooled estimate of syphilis-HIV co-infection was also assessed. Consequently, the overall pooled prevalence was 0.80% (95% CI, 0.60–1.01%). A comparable result (0.73%) was obtained from Republic of Congo [37]. While a study in Tanzania reported lower (0.3%) prevalence [35], another study from Rwanda indicated higher (1.2%) prevalence [38] of syphilis-HIV co-infection relative to the findings of this study. The difference in prevalence of syphilis-HIV co-infection might be attributed to the variation in level of implementation and integration of STI prevention and control measures in different countries.

Besides, the association between pregnant women with and without previous history of multiple sexual partner and syphilis sero-positivity was measured in this study. Accordingly, pregnant women with previous history of multiple sexual partners were 2.98 times more likely to get syphilis infection compared to women without such partner. This was consistent with previously established evidences [39–42] and could be attributed to the fact that people with multiple sexual partner has higher risk of getting STI including syphilis. This study also showed that pregnant women with previous history of STI were 4.88 times at higher risk of developing syphilis relative to pregnant women without such history. This is in line with the findings from Malawi [43] and China [44]; this could be partly due to lack of behavioral change and other prevention interventions that resulted in maintaining risky behaviors among women who had history of previous STI.

On the other hand, this meta-analysis also demonstrated that married women had 63% lower risk of developing syphilis compared to those who are not cohabiting (single, widowed, divorced). This was comparable with earlier studies conducted in rural Tanzania [45] and three sub-Saharan countries [46]. This might be due to the tendency of non-cohabiting women to practice high-risk sexual behaviors like having multiple sexual partners. Partly, this could be because of women in ANC are sexually active age groups, suggesting that if they are non-cohabiting they may have high-risk sexual behaviors.

### Limitations

Given the difficulty of syphilis diagnosis, most of the included studies used treponemal diagnostic test alone which may impact the prevalence report. Some regions in the country were not represented in this study due to lack of established original studies in the area. Furthermore, all included studies were facility based. Thus, interpretation of findings has to be with due consideration of these limitations.

## Conclusions

This review provides evidence of relatively high prevalence of syphilis among pregnant women. This study also assessed the burden of syphilis-HIV co-infection and determinants of syphilis sero-reactivity in Ethiopia. Therefore, it is recommended to further ramping up of current intervention measures, like routine screening of all ANC women and integration of syphilis testing and treatment to the already established HIV prevention program in the country. Further, nationwide studies involving all regions are needed to assess the magnitude and determinant factors for syphilis among pregnant women in Ethiopia.

## Supplementary Information

**Additional file 1: **PRISMA-P 2015 Checklist.

## Data Availability

Any data related to this manuscript will be accessible by requesting the corresponding author.

## Abbreviations

ANC: Antenatal care
CI: Confidence interval
HIV: Human immunodeficiency virus
ICS test: Immuno-chromatography test
OR: Odds ratio
RPR: Rapid plasma reagin
SNNP: Southern Nations Nationalities and Peoples of Ethiopia
STI: Sexually transmitted infection
TPHA: Treponema pallidum hemagglutination assay
VDRL: Venereal diseases research laboratory
